# Opening of a Large New Wing

**Published:** 1914-08-01

**Authors:** 


### THE BRISTOL GENERAL HOSPITAL.

### Opening of a Large New Wing.

On Monday afternoon the President of the Bristol General Hospital, Mr. George A. Wills, opened the large new wing which has been added to the institution at a cost of £45,000. The whole of the money was found—thanks to an offer to provide £2,000 which the furnishing will cost—at the last moment by an anonymous friend, and this enabled the Treasurer, Mr. Herbert M. Baker, to announce that the wing was opened free of debt so far as capital expenditure is concerned. It was a very gratifying, if not altogether surprising, accompaniment of the opening.

The project was taken in hand through a gift of £10,000 by the late Mrs. Proctor Baker, whose husband was once President of the hospital. Her gift was for a maternity ward, but other needs were pressing, and the Committee decided to embody this provision in the ambitious scheme which was completed on Monday. When the fund was opened money came in very rapidly, and the Committee were soon in a position to go on with the building, and to embody in it equipment which makes the hospital much more comprehensive in its scope.

In addition to the maternity and female sick wards, and ambitious accommodation for nurses and staff, a fine dental department is provided. Another prominent and important feature is the space set apart for clinical and pathological laboratory work. The situation of the building is most advantageous, ensuring light, air, and sun to the utmost extent. The large wards open out on to two covered sun-balconies, and the flat roof is to be laid out as a roof-garden, which commands an extensive view of the city and neighbourhood. The whole of the scheme has been admirably planned and carried out, and the influential citizens who were present at the opening could hardly fail to be struck with the way in which the interior arrangements met the most modern requirements of hospital equipment. All told, the number of beds is now 250.

The President gave a brief history of the hospital, founded in 1832, and extended from time to time from the original thirty beds to over 200, and now to 250. They now rejoiced that they had an institution up to date, and fitted with all that modern science demanded. He expressed high appreciation of the staff, of the Matron, Miss Densham, and those under her, who did their work with devotion and wholeheartedness. He expressed the hope that that and other hospitals would be liberally supported.

The Treasurer, Mr. Baker, said that when Mrs. Proctor Baker gave £10,000 for a maternity ward the Committee decided it should be the nucleus of a great effort. They realised the importance of a dental department, and the need of an enlarged pathological department and clinical laboratory, as well as improved accommodation for staff and nurses. In a few weeks the £10,000 grew into £43,000. There was, he said, no institution like their new dental department in that part of the country. It was fitted up in the most perfect way from all points of view. He mentioned that no less than £26,000 had been given by friends who had died since the commencement of the scheme. He emphasised the fact that the enlargement would entail greater annual expense, and the need of more liberal income from annual subscriptions.

Dr. Michell Clarke spoke for the honorary staff, and said that the accommodation was urgently needed in order to put the hospital in a position to carry on its work satisfactorily and do first-class work. Alluding to the feeling abroad that the Insurance Act would mean that the demands on hospitals would not be so great, he said he wished to correct that view. He was inclined to think, and their experience, so far, went to show, that the demands on their beds would be greater than before, certainly not less, in consequence of the Insurance Act. That Act at present made no provision for persons who required treatment in bed by skilled nurses or by special surgical or medical treatment. Moreover, as under the Act people were seen much earlier than they were before, he believed a beneficent effect of the Act would be that diseases would be discovered earlier than before, and that a larger number would be sent there and to similar institutions for treatment. Therefore, he did not look forward to any curtailment of the demand upon their beds. He hoped that the voluntary system that had been carried out in the great hospitals throughout the country would still be carried on for a great many years. He felt perfectly sure that no compulsory system would ever be as efficient as the work which was being done as a matter of free-will and of love. He spoke of the rapid advances of the medical and surgical arts, with the reduction in mortality and suffering, and said in that great work perhaps the largest share had been taken by the voluntary hospitals. They had attracted from the first the best minds of the medical profession to work in them, and they had afforded opportunities for research and study which were not obtainable elsewhere.

An important feature of the extension was the opening of the pathological and chemical laboratory. They had a good laboratory before, with a special pathologist, but that work had grown so much that they required a larger laboratory. That side of their work was becoming increasingly important, because it enabled them more completely and adequately to study disease and carry on the research work for its prevention. That was a work which would be more and more incumbent on great hospitals. He had a word or two to say on the idea, that is met with sometimes, that patients in a hospital are the subjects of experiments—he meant experiments in a doubtful sense. Every patient, to a greater or less extent, whether in or out of a hospital, was the subject of experiment, but to say that patients in a hospital were experimented on in a way they would not be outside a hospital was not true. There was nothing carried on there or in any good hospital which would not be equally carried out in private practice.

He referred to the dental department, and said they had a large one because they all now recognised the very great importance of diseases of the teeth in their relation to the general health and as the causes of other diseases. A dental department, such as that, put dental treatment within the reach of the working classes, and was a legitimate extension of the proper sphere of work of the hospital, and he trusted it would increase its usefulness.

The President opened the door with a handsome gold key, the gift of the architects, Messrs. Oatley and Lawrence, and then the large number of visitors inspected the building, which is an important addition to the hospital equipment which the city now has at the General Hospital, the Royal Infirmary, the Children's Hospital, and the minor institutions for medical relief.

